# Time-dependent cytokines changes in ultra-rush wasp venom immunotherapy

**DOI:** 10.1038/s41598-023-37593-0

**Published:** 2023-06-29

**Authors:** W. Urbańska, L. Szymański, M. Ciepelak, A. Cios, W. Stankiewicz, E. Klimaszewska, Krystyna Lieto, Rafał Skopek, A. Chciałowski, S. Lewicki

**Affiliations:** 1 Department of Infectious Diseases and Allergology, Military Institute of Medicine, National Research Institute, Szaserów 128, 04-141 Warsaw, Poland; 2 Department of Molecular Biology, Institute of Genetics and Animal Biotechnology, Polish Academy of Sciences, Postępu 36A, 05-552 Magdalenka, Poland; 3 Department of Microwave Safety, Military Institute of Hygiene and Epidemiology, Kozielska 4, 01-163 Warsaw, Poland; 4 Department of Hematological and Transfusion Immunology, Institute of Hematology and Transfusion Medicine, 14 I. Gandhi St., 02-776 Warsaw, Poland; 5 Faculty of Health Sciences, The Mazovian State University in Płock, Generała Jarosława Dąbrowskiego 2, 09-402 Płock, Poland; 6 Faculty of Medical Sciences and Health Sciences, Kazimierz Pulaski University of Technology and Humanities in Radom, 26-600 Radom, Poland; 7 Institute of Outcomes Research, Maria Sklodowska-Curie Medical Academy, 00-001 Warsaw, Poland

**Keywords:** Immunology, Chemokines, Cytokines, Immunotherapy

## Abstract

Venom immunotherapy (VIT) represents a potential therapeutic approach for the management of venom allergies, aiming to modify the immune response to venom allergens and enhance its precision. Previous studies have demonstrated that VIT induces a shift in T helper cell responses from Th2 to Th1, characterized by the production of IL-2 and interferon-gamma by CD4^+^ and CD8^+^ cells. In order to explore long-term pathways following VIT treatment and verify potential new outcomes, the serum concentrations of 30 cytokines were assessed in a cohort of 61 patients (18 control, 43 study group) exhibiting hypersensitivity to wasp venom. Cytokine levels were measured at 0, 2, 6, and 24 weeks after the initiation phase of VIT in the study group. The present study found no significant alterations in the levels of IL-2 and IFN-γ in the peripheral blood following VIT. However, a noteworthy finding was the substantial increase in the concentration of IL-12, a cytokine capable of promoting the differentiation of Th0 cells into Th1 cells. This observation supports the involvement of the Th1 pathway in the desensitization process induced by VIT. Additionally, the study revealed a significant rise in the levels of IL-9 and TGF-β after VIT. These cytokines may play a role in the generation of inducible regulatory T (Treg) cells, indicating their potential importance in the immune response to venom allergens and the desensitization process associated with VIT. Nevertheless, further investigations are required to comprehend the underlying mechanisms driving the VIT process comprehensively.

## Introduction

Hymenoptera venom allergy (HVA) represents a severe allergic condition characterized by a notable prevalence of severe anaphylactic reactions, occasionally leading to fatalities. The estimated annual mortality rate ranges from 0.03 to 0.45 per one million individuals^1^. Anaphylactic reactions triggered by Hymenoptera stings can result in life-threatening anaphylactic shock, occurring within a median time of 15 min and necessitating prompt administration of intramuscular epinephrine. The gold standard for diagnosis involves conducting a skin prick test, intradermal test, or evaluating the concentration of Hymenoptera serum-specific IgE (sIgE)^2^. In Poland, Hymenoptera venom allergy is predominantly associated with wasp stings, which often exhibit severe clinical courses in sensitized patients^3^. While avoiding areas with high concentrations of these insects is the primary method of preventing wasp stings, it may not be feasible for certain professions, such as foresters or gardeners. Wasp venom immunotherapy (VIT) serves as a solution for these patients.

VIT entails the administration of insect venom preparations through a series of subcutaneous injections. The current guidelines from the American Academy of Allergy and Clinical Immunology and the European Academy of Allergy and Clinical Immunology recommend VIT for patients with a history of systemic sting reactions^4^. The time required to reach the maintenance dose varies depending on the protocol used, which can be conventional, rush, or ultra-rush. In our department, we employ the ultra-rush method as the initial approach, followed by administrations at 4-week intervals for a duration of 3–4 years. VIT is effective in approximately 80–95% of patients with bee venom and Vespula venom allergy^5^.

VIT acts on the immune system of sensitized patients through various complex mechanisms. Repeated exposure to escalating doses of an allergen can reverse hypersensitivity and confer lifelong tolerance to venoms. During VIT, a shift from a Th2 to Th1 immune response is observed, which can be monitored through cytokine profiles^6^. Moreover, in our previous study, we noted an increase in IL-10 release from immunosuppressive regulatory T cells (Treg), which is crucial for inducing allergen tolerance^7^. IL-10 suppresses allergen-specific Th2 response, limits Th1, NK cells, and their cytolytic activity, induces IgG4 production by tissue and lymph nodal B cells, inhibits mast cells, basophils, and eosinophil recruitment and activation^8^. Treg cells have also been associated with the production of transforming growth factor beta (TGF-β), a potent cytokine that suppresses the immune response. It has been repeatedly proven that TGF-β inhibits the proliferation of various types of cells, i.e., hematopoietic, endothelial, and lymphatic cells 9. An accumulating body of evidence may suggest that methods for generating the Treg cell population may improve the efficacy of VIT.

In light of the current knowledge, IL-10 and TGF-β play established roles in elucidating the immunosuppressive mechanism of VIT, certain aspects of the VIT mechanism remain unclear, such as the involvement of other cytokines like IL-9 and different TGF-β isoforms. Thus, the objective of our study was to investigate the role of 30 cytokines, including interleukins, chemokines, growth factors, and IL-1 receptor antagonists, in the development of immunotolerance to wasp venom.

## Materials and methods

### Patients

The study includes 61 patients from the Department of Infectious Diseases and Allergology, Military Institute of Medicine (Warsaw, Poland) with overreaction to wasp venom. The study cohort comprised individuals aged between 20 and 70 years, with a median age of 50 years. Participants were devoid of any chronic ailments that might contraindicate the administration of immunotherapy. A limited number of cases exhibited untreated hypertension without the use of beta-blockers. Individuals with active cancer, autoimmune diseases in their active phase, or acquired immunodeficiency syndrome (AIDS) were deemed ineligible for study inclusion. Additionally, pregnant women were also excluded from participation. Participants were not stung by *Hymenoptera* within the study period. The diagnosis was confirmed through the skin and intradermal tests as well as sIgE measurements in patients with a positive history of allergic reactions after stings. Eighteen patients (Müller's grades I and II) were qualified for to control group, and 43 patients (Müller's grades III and IV) were in the study group. The demographic characteristic and IgE analysis of the patients was described previously^7^. The study's general exclusion criteria included cardiovascular and oncological diseases and medication with systemic drugs that reduced immune system functions. The study was performed according to the Bioethics Committee resolution in the Military Institute of Medicine (No. 130/WIM/2018). All patients signed an informed consent form, and those undergoing VIT received oral antihistamines as a pretreatment before the ultra-rush induction phase and during the maintenance treatment.

### Desensitization

Desensitization was performed only in the study group, as previously described in detail^7^. Briefly, the wasp venom (Venomenhal, 120 µg of wasp venom/vial) was given using the ultra-rash protocol (0,1 μg, 1 μg, 10 μg, 20 μg, 30 μg, and finally 40 μg in the 30 min intervals—total 101.1 μg in induction phase—point "0"). Then, two weeks later, and then every four weeks, 100 μg of the Venomenhal was administrated.

### Blood collection

Blood was collected immediately before Venomenhal injection (0, 2, 6, and 24 weeks after the induction phase) from the vein to the tube for serum preparation. After collection, blood was immediately transported to the laboratory, the blood was centrifuged (20 min, 2000×*g*), and the serum was collected and frozen (− 80 °C) for further analysis.

### Cytokines analysis

Serum cytokines concentration: interleukins-1b, -2, -4, -5, -6, -7, -8 (CXCL8), -9, -10, -12, -13, -15, -17A, TNF-α, IFN-γ, TGF-β1-3) chemokines (MIP-1a (CCL3), MIP-1b (CCL4), MCP-1, RANTES (CCL5), Eotaxin (CCL11), IP-10) growth factors (G-CSF, GM-CSF, PDGF, bFGF and VEGF) and receptor antagonist of IL-1 were measured using Bio-Plex Pro TGF-β Panel 3-Plex (Bio-Rad) and Bio-Plex Pro Human Cytokine Grp I Panel 27-Plex (Bio-Rad, Poland). Data analysis was performed on a Bio-Plex 200 system in conjunction with Bio-Plex Manager version 6.1.1 using a 5-parameter (5-PL) nonlinear logistic regression curve fit model (Bio-Rad). As per Bio-Rad Bio-Plex Multiplex Immunoassay recommendations, the assay's sensitivity was defined as analyte concentration corresponding to the median background fluorescence intensity (MFI) plus two standard deviations (SD) of the mean background MFI.

### Statistical analysis

All results are presented as the mean ± SD. The data distribution was evaluated using the Shapiro–Wilk test. Statistical evaluation of the results was performed using the Kruskal–Wallis test with Dunn's multiple comparison test. Results at p < 0.05 were considered statistically significant. GraphPad Prism software (version 9.4.1; GraphPad Software, Inc., La Jolla, CA, USA) was used for statistical calculations.

### Institutional review board statement

The study was conducted according to the guidelines of the Declaration of Helsinki, and approved by Ethics Committee of Military Institute of Medicine, Warsaw Poland, resolution No. 130/WIM/2018.

## Results

In order to present the analysis more precisely, we divided the analyzed factors into six groups: Th1, Th2, Th9, Th17, Treg cytokines, and growth factors.

### Th-1 pathway

Th1 cytokines are crucial in the immune response against intracellular viruses and bacteria. In addition, they are responsible for promoting cellular defense mechanisms.

IL-12 and IFN-γ are key factors that drive the differentiation of naive Th0 lymphocytes into Th1 cells. Stimulated Th1 lymphocytes synthesize and secrete mainly IL-2, -12, TNF-α, and IFN-γ. The Th1 pathway is also influenced by other members of the IL-2 family, such as IL-7 and IL-15, as well as chemokines, including CCL3, CCL4, CCL5, CCL11, and CXCL10, which interact with Th1-specific receptors, namely CCR5 and CXCR3. The primary effector cells of the Th1 pathway include CD4^+^ T cells that secrete IFN-γ, CD8^+^ T cells, B cells producing IgG antibodies, and macrophages.

**Figure 1:**
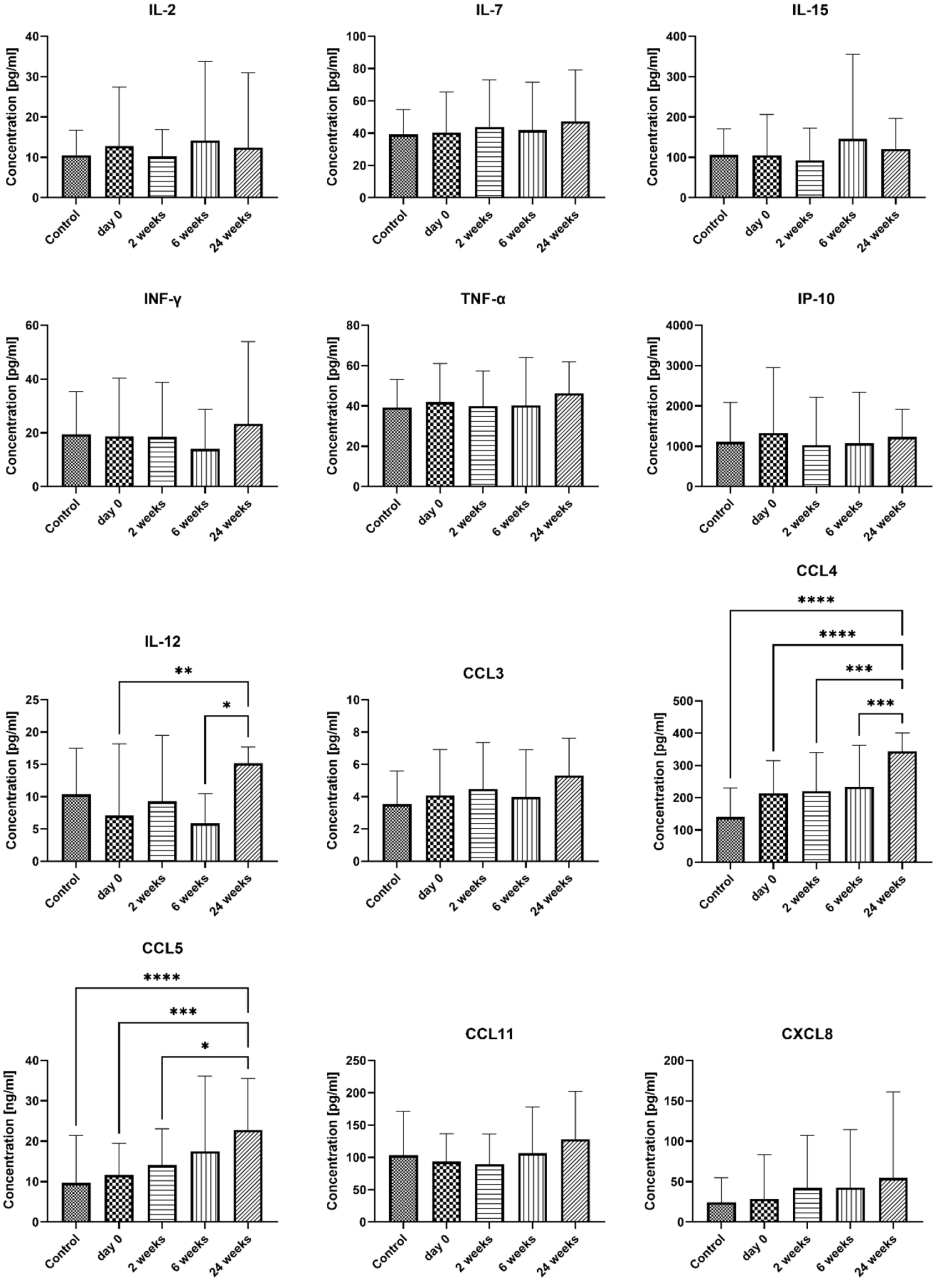
The serum concentration of Th1-related cytokines and chemokines. Blood was collected at day 0 (control and examined group) and after 2, 6, and 24 weeks of VIT (examined group only). Results are presented as mean ± SD. *Level of difference.

At the beginning of the study, there were no significant differences in the serum concentrations of Th1-related cytokines and chemokines between the study group and the control group. Moreover, no changes were observed in the concentrations of cytokines belonging to the IL-2 family (IL-2, IL-7, IL-15), the main antiviral cytokine (IFN-γ), the proinflammatory cytokine TNF-α, and the chemokine IP10 in the serum of patients undergoing VIT at selected time points (0, 2, 4, and 24 weeks after the induction phase). However, a significant increase in IL-12 concentration was found in the serum of patients from the study group after 24 weeks following the first VIT treatment (7.08 ± 11.11 pg/ml and 15.20 ± 2.477 pg/mg, p = 0.002, before treatment and 24 weeks after the first dose, respectively). Among the Th1-related chemokines, CCL3, CCL11, and CXCL8 showed no differences between the control and study groups before treatment. However, there was considerable variability in CXCL8 concentration in the examined group prior to VIT (ranging from 0.62 to 2218.38 pg/ml). CCL4 and CCL5 did not differ between the studied groups; however, their concentrations increased over time, with the most significant differences observed at 24 weeks (CCL4: 213.7 ± 101 pg/ml and 344 ± 56.78 pg/ml, p < 0.0001; CCL5: 11.65 ± 7.802 ng/ml and 22.75 ± 12.79 ng/ml, p = 0.0004; before treatment and 24 weeks after the first dose, respectively). Results are presented in Fig. 1.

### Th2 pathway

Th2 cells play a crucial role in defending the human body against extracellular parasites and are involved in anti-inflammatory responses. The development of Th2 cells from naive Th0 cells is coordinated by IL-4. Th2 cells primarily secrete IL-4, IL-5, and IL-13, which are essential for humoral immunity. Upon antigen stimulation, activation of the Th2 pathway leads to the production of IgE antibodies. In addition to their role in combating extracellular parasites, dysregulation of the Th2 pathway is associated with the development of type I hypersensitivity reactions, such as allergies to wasp venom. The primary effector cells involved in the Th2 pathway include CD4^+^ T cells (producing IL-4, IL-5, and IL-13), B cells producing IgE antibodies, eosinophils, basophils, and mast cells.

**Figure 2:**
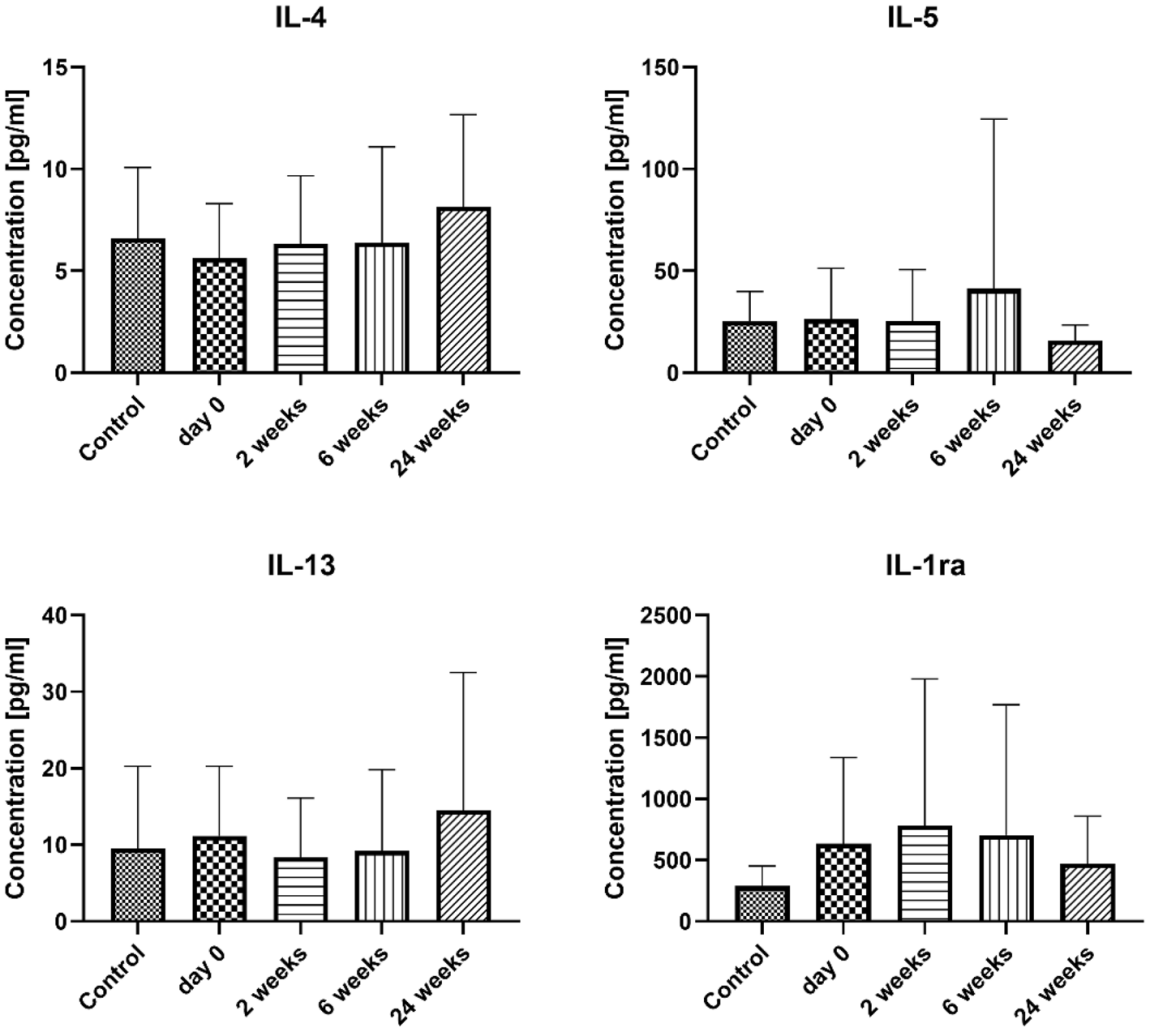
The serum concentration of Th2-related cytokines and chemokines. Blood was collected at day 0 (control and examined group) and 2, 6, and 24 weeks after VIT (examined group only). Results are presented as mean ± SD. *Level of difference.

No significant changes were observed in the serum concentrations of IL-4, IL-5, and IL-13 between the control and study groups. Furthermore, these cytokine levels did not show any significant changes over time in the serum of patients undergoing VIT. Similarly, the antagonist of the IL-1 receptor did not exhibit any abnormalities in the serum of the examined groups (Fig. 2). Interestingly, we observed similar trends in the concentration of IL-1β and the IL-1 receptor antagonist (IL-1ra) at the studied time points; however, these changes were not statistically significant. Results are presented in Fig. 2.

### Th9 pathway

IL-9 secreting Th9 cells play a role in various immune processes such as allergic responses, tumor suppression, immunity against pathogens, and immune-mediated diseases. The development of Th9 cells is regulated mainly by IL-4 and requires the activation of STAT6, GATA3, and IRF4, but IL-4 alone leads to the formation of Th2 cells. For the differentiation of T cells into the Th9 subpopulation, IL-4 signaling must be accompanied by the TGFβ superfamily signaling. Interestingly, stimulation of naïve T cells with the TGFβ, in the absence of IL-4, leads to the development of inducible Treg cells. The presence of Th9 cells (CD4^+^IL-9^+^IL-13^−^IFNγ^−^) was found in the blood of allergic patients, while in healthy individuals, this population is scarce.

No significant changes in the concentration of TGFβ3 have been observed. TGFβ1and TGFβ2 did not differ between studied groups, but their concentrations significantly increased 24 weeks after the initiation of the treatment (TGFβ1: 6.395 ± 5.350 ng/ml and 46.99 ± 46.76 ng/ml, p = 0.0004; TGFβ2: 231 ± 104.8 pg/ml and 714.2 ± 546.7 pg/ml, p = 0.0007, before treatment and 24 weeks after the first dose, respectively).

**Figure 3:**
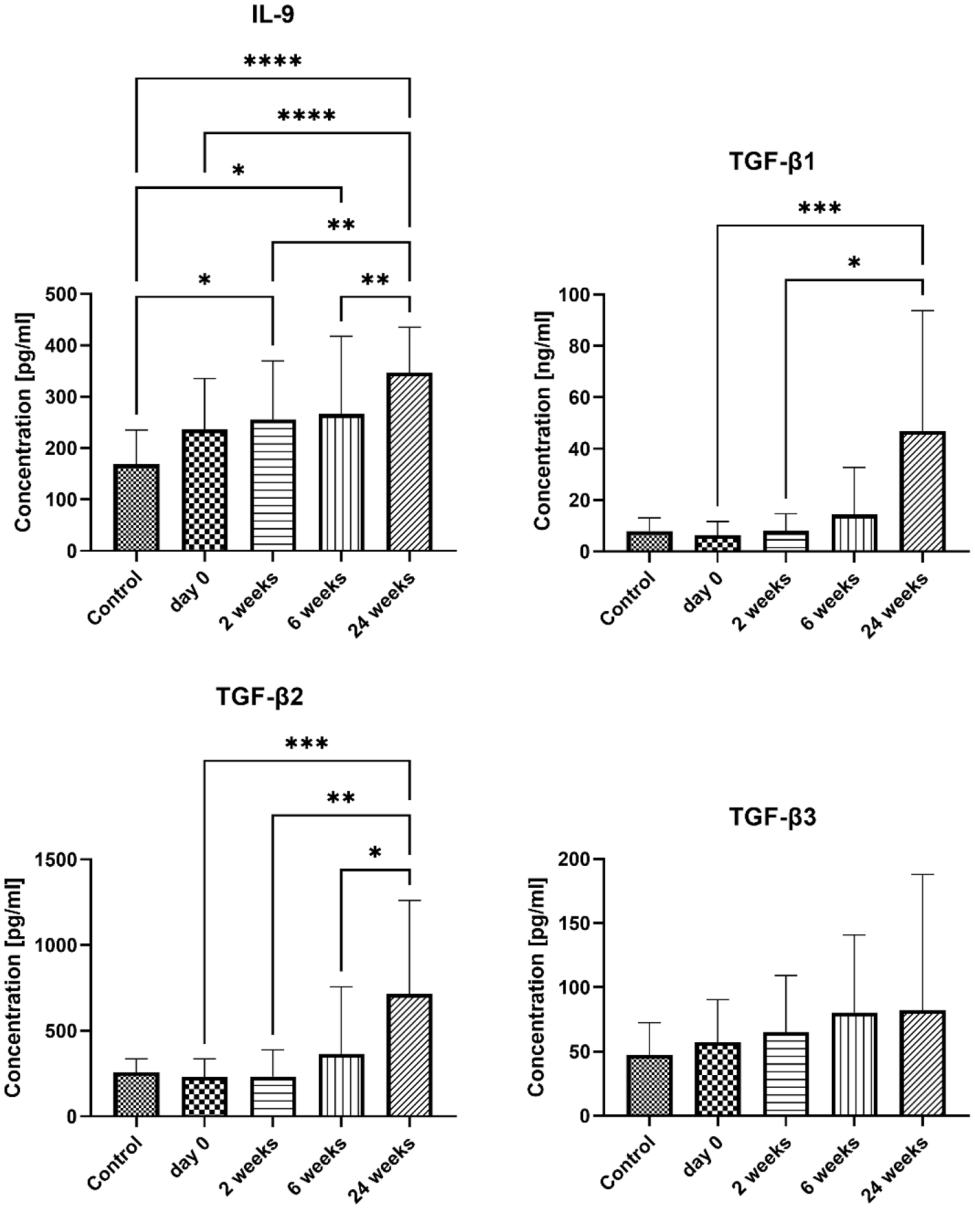
The serum concentration of Th9-related cytokines and chemokines. Blood was collected at day 0 (control and examined group) and 2, 6, and 24 weeks after desensitization (examined group only). Results are presented as mean ± SD. *Level of difference.

IL-9 concentration did not differ between the study and control group, but it increased over time, with the highest and most significant differences at 24 weeks (347.1 ± 88.02 pg/ml) after initiation of the treatment compared to the control (168.3 ± 66.98 pg/ml, p < 0.0001) and the treatment initiation (236.6 ± 99.16 pg/ml, p < 0.0001). Results are presented in Fig. 3.

### Th17 pathway

Th17 cells are a type of proinflammatory T helper cells that secrete IL-17. They play a crucial role in defending against extracellular pathogens and mediating innate immunity. However, they are also implicated in the development and progression of inflammatory diseases like rheumatoid arthritis and psoriasis. The differentiation of Th17 cells requires the presence of TGFβ, along with either IL-6 or IL-21, which activate STAT3 signaling. The pathogenicity of Th17 cells is also influenced by the specific isoform of TGFβ, with Th17 cells induced by TGF‐β1 being less pathogenic, while those induced by TGF‐β3 are more pathogenic.

**Figure 4:**
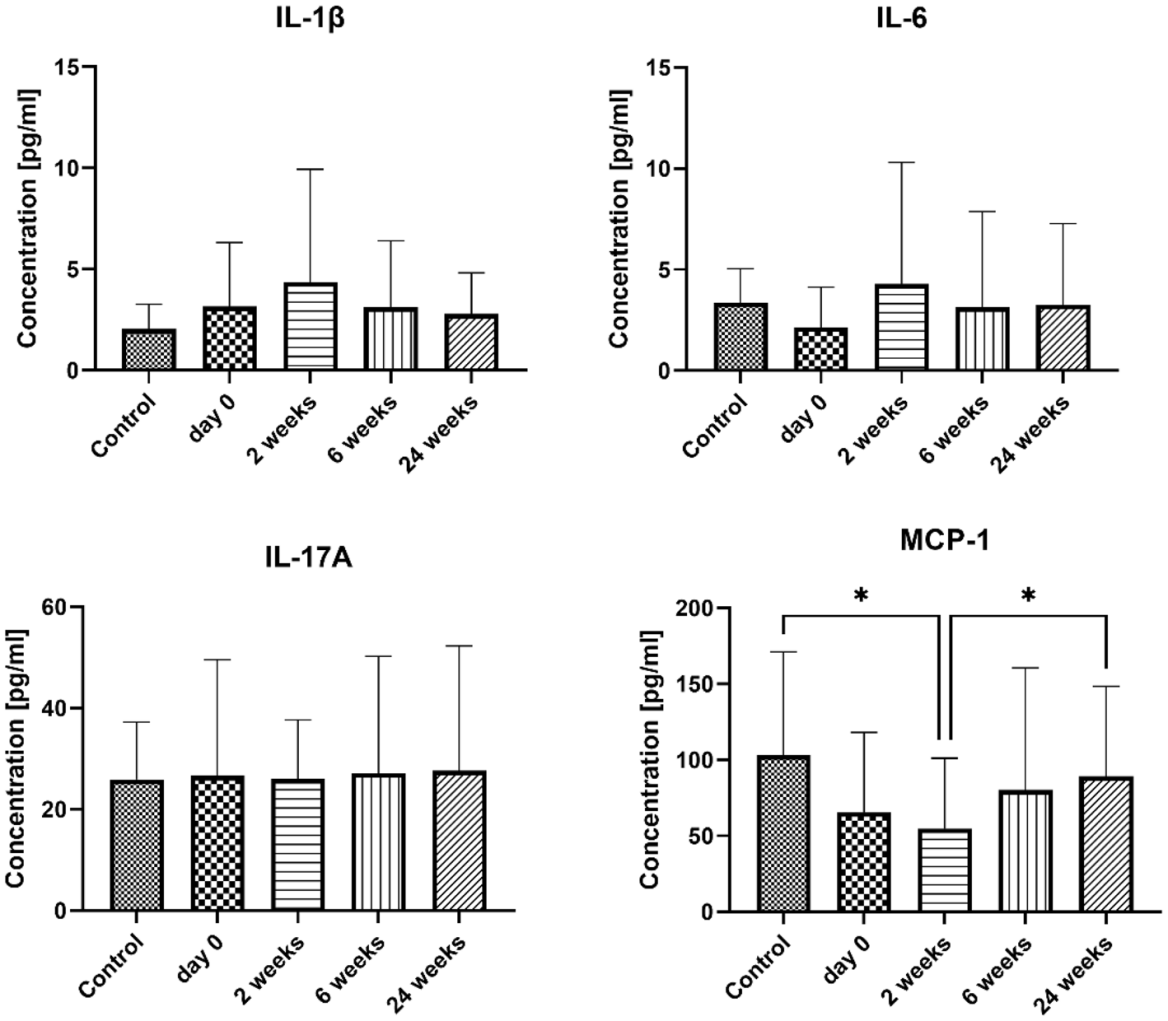
The serum concentration of Th17-related cytokines and chemokines. Blood was collected at day 0 (control and examined group) and 2, 6, and 24 weeks after VIT (examined group only). Results are presented as mean ± SD. *Level of difference.

In the present study, no significant changes in the concentration of Th17-related cytokines, including IL-1β, IL-6, and IL-17A, except for MCP-1. The MCP-1 levels decreased two weeks after the initiation of treatment compared to the control group (54.75 ± 46.30 pg/ml and 103.1 ± 67.94 pg/ml, p = 0.0308, respectively). However, MCP-1 levels subsequently increased again at 6 and 24 weeks and were not significantly different from the control group. Results are presented in Fig. 4.

### Treg pathway

Regulatory T cells (Tregs) are a specialized subset of CD4^+^ T cells that express the transcription factor Foxp3. They have a unique function in suppressing the immune response, promoting self-tolerance, and maintaining immune homeostasis. Tregs play a crucial role in preventing autoimmune reactions by inhibiting T cell proliferation and cytokine production. There are two main subpopulations of Tregs: natural Tregs (nTregs) and induced Tregs (iTregs). nTregs develop during T cell maturation in the thymus through self-activation of the T cell receptor, which leads to the expression of Foxp3. These mature nTreg cells primarily maintain self-tolerance in the periphery. On the other hand, iTregs arise from CD4^+^ conventional T cells in peripheral lymphoid organs and focus on regulating the immune response against foreign antigens. The differentiation of Tregs is influenced by key factors such as IL-2 and TGFβ, while the secretion of IL-10 is one of the multiple mechanisms employed by these cells to suppress the immune system.

**Figure 5:**
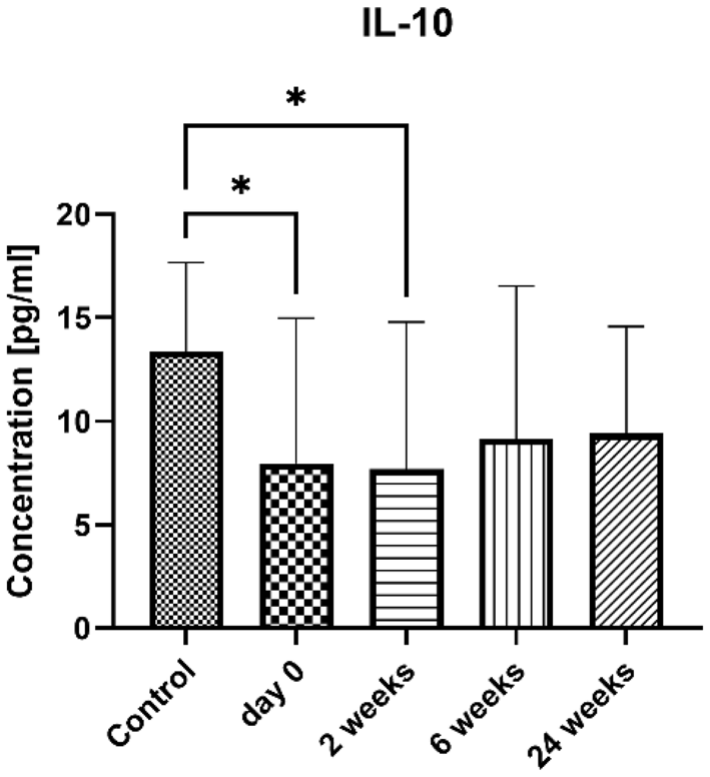
The serum concentration of Treg-related cytokines and chemokines. Blood was collected at day 0 (control and examined group) and 2, 6, and 24 weeks after VIT (examined group only). Results are presented as mean ± SD. *Level of difference.

In this study, we observed a statistically significant difference in the concentration of IL-10 between the control group (13.37 ± 4.316 pg/ml) and the study groups before (7.976 ± 6.999 pg/ml, p = 0.0355) and 2 weeks after (7.693 ± 7.095 pg/ml, p = 0.0197) the initiation of treatment. However, during the course of the VIT, the IL-10 concentration increased at 6 weeks (9.135 ± 7.384 pg/ml) and 24 weeks (9.428 ± 5.149 pg/ml) after treatment initiation, and the difference compared to the control group became statistically insignificant (p > 0.1). Results are presented in Fig. 5.

### Growth factors

Initially, growth factors were described as soluble proteins that influence cell growth. However, over time, the definition expanded to include molecules that regulate cell differentiation, proliferation, and mitosis. Furthermore, growth factors can exert their stimulatory effects through autocrine, endocrine, and paracrine mechanisms. Typically, they exert their signaling function by binding to specific receptors on the cell surface, transmitting specific instructions to intracellular components, and ultimately leading to altered gene expression.

**Figure 6:**
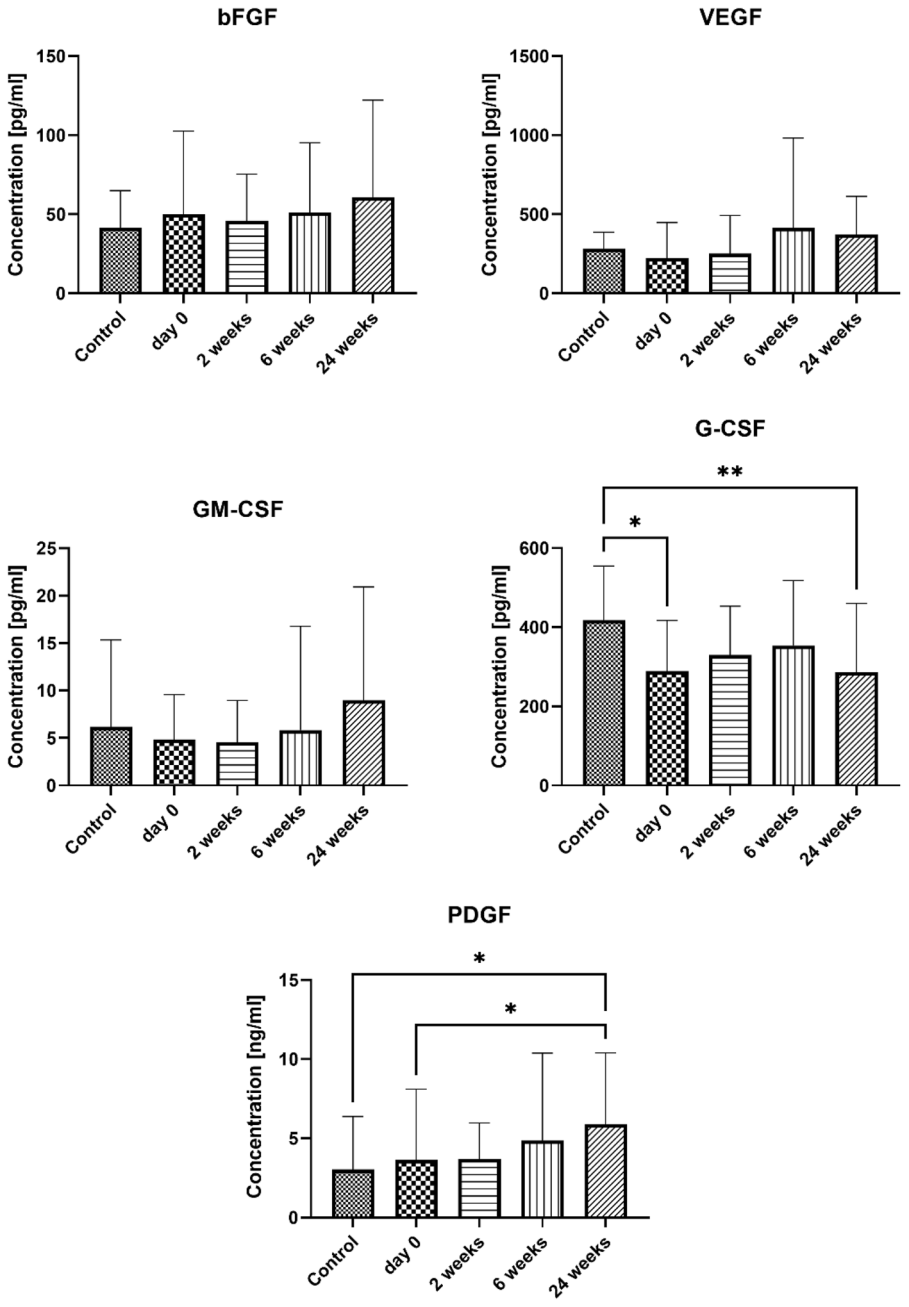
The serum concentration of selected growth factors. Blood was collected at day 0 (control and examined group) and 2, 6, and 24 weeks after VIT (examined group only). Results are presented as mean ± SD. *Level of difference.

In the present study, we did not observe any significant differences in the concentrations of bFGF, VEGF, and GM-CSF between the control and the study groups. However, the concentration of G-CSF in the serum of patients was significantly lower before the initiation of VIT compared to the control group (289.2 ± 128.3 pg/ml and 417.9 ± 136.6 pg/ml, p = 0.0189, respectively). During the course of VIT, the concentration of G-CSF increased at two weeks (329.8 ± 123.3 pg/ml) and six weeks (353.9 ± 164.6 pg/ml) after treatment initiation, reaching levels statistically indistinguishable from the control group (p > 0.4). However, at 24 weeks after the initiation of VIT, the concentration of G-CSF was significantly lower than in the control group (287 ± 173.4 pg/ml and 417.9 ± 136.6 pg/ml, p = 0.0066, respectively). The concentration of PDGF did not differ between the studied groups, but it significantly increased at 24 weeks (5.891 ± 4.501 ng/ml) after the initiation of treatment compared to the control group (3.029 ± 3.346 ng/ml, p = 0.0191) and the group before the initiation of VIT (3.634 ± 4.475 ng/ml, p = 0.0099). Results are presented in Fig. 6.

## Discussion

The protection of an organism against foreign substances is essential for its survival in the natural environment. The human immune system encompasses two types of immune responses, namely innate and adaptive responses. The adaptive response is highly specific and effective in eliminating invading pathogens. However, to avoid damaging the body's own molecules, it is crucial for the immune system to distinguish between foreign substances and self. This ability to discriminate is a fundamental aspect of the adaptive immune system. Unfortunately, there are instances when the immune system fails to make this distinction, leading to autoimmune reactions or misidentifies harmless substances as threats, resulting in hypersensitivity reactions. Several factors, including genetics, environmental influences, and antigen-presenting cells, contribute to the learning process of adaptive immune cells in recognizing foreign substances. Cytokines play a significant role in regulating this process. Upon antigen interaction, Th cells differentiate into various subpopulations, such as Th1, Th2, Th17, Th19, and Th22, guided by the cocktail of cytokines they encounter^10^. Once differentiated, Th cells influence the responses of other immune cells.

Allergic reactions, also known as type I hypersensitivity, involve an immune response triggered by the presence of an antigen. Th2 lymphocytes primarily mediate this response, aiming to eliminate the threat. In this process, B-cells produce IgE, which binds to mast cells and basophils. The secretion of IL-4 and IL-5 plays a regulatory role in this process, with IL-5 specifically promoting the production of eosinophils and basophils in the bone marrow^11^.

Immunotherapy involves the deliberate modification of the immune system's response to an antigen to enhance its precision. Previous studies have provided evidence that individuals with allergic rhinitis or those undergoing allergen immunotherapy exhibit significantly reduced levels of IL-4 compared to untreated individuals, and these levels resemble those observed in non-allergic individuals. This indicates the effectiveness of immunotherapy in modifying the immune response to allergens^12^. Previous research has shown that following VIT, levels of IL-4 are significantly decreased at 15 and 45 days post-treatment. However, these cytokine levels were measured in the supernatant of isolated peripheral blood mononuclear cells (PBMCs) cultured for six days in RPMI1640 medium^13^. In the current study, VIT did not affect the levels of IL-4, IL-5, and IL-13 in the peripheral blood. There are two potential explanations for this disparity. Firstly, the cytokine levels were only measured at specific time points following VIT treatment, rather than continuously monitoring their changes. Secondly, the immune stimulation induced by venom allergens may not be as potent as that caused by inhaled or food allergens, indicating that maintaining elevated levels of these cytokines in the bloodstream would not be necessary or beneficial for the body. In fact, even in cases of allergic rhinitis, serum levels of IL-4, IL-5, and IL-13 did not significantly exceed those of the control group^14^.

Previous research has shown that immunotherapy for wasp venom triggers a shift in T helper cell responses from Th2 to Th1. This shift is characterized by the secretion of IL-2 and interferon-gamma by CD4^+^ and CD8^+^ cells, as observed in the supernatants of stimulated PBMCs obtained from desensitized patients^15,16^. However, in this study, there were no significant changes in the concentration of IL-2 and IFN-γ in the peripheral blood. Instead, the study found a significant increase in the concentration of IL-12, which is a cytokine known to induce the differentiation of Th0 cells into Th1 cells. Morgan et al. also observed this phenomenon, but only in the culture of monocytes isolated from allergic patients exposed to yellow jacket venom^17^. This supports the role of the Th1 pathway in the desensitization process induced by VIT treatment. Additionally, the study documented a time-dependent increase in the serum concentration of the chemokines CCL4 and CCL5, which are produced by various inflammatory cells. Notably, CCL4 and CCL5 contribute to the recruitment of Th1 cells^18^. It is worth noting that CCL5 levels did not significantly increase in the serum after the first day of VIT with wasp venom but did increase after VIT with bee venom^19^. Both CCL5 and CCL4 activate CCR5, which is a receptor expressed on T follicular helper cells (CXCR5^+^ FoxP3^+^ cells). These cells play a role in B-cell maturation and the switching of immunoglobulin classes and are also capable of suppressing T- and B-cell responses^20^. The increase in IL-12, CCL5, and CCL4 in the serum after VIT suggests that the Th1 pathway is involved in long-term changes, including a switch in the production of B-cell wasp-specific antibodies from IgE to IgG.

In this study, VIT was found to significantly increase the levels of two types of TGF-β (1 and 2). GF-β is known to contribute to the development of three distinct subclasses of T helper cells, namely Th9, Th17, and Treg cells. Th9 cells arise from Th0 cells and are characterized by the secretion of IL-9, IL-10, and IL-21^21^. IL-9 has been linked to the accumulation and activation of mast cells, the stimulation of mucin production, the increased chemotaxis of eosinophils, and the stimulation of IgE production by B-cells^22^. Elevated levels of IL-9 have been found in patients with inhaled^23^, food^24^, or contact dermatitis allergies^25^. In fact, an anti-interleukin-9 antibody is currently being tested as a potential adjuvant to immunotherapy in a mouse model of allergic rhinitis^26^. Studies utilizing a mouse model of experimental allergic airway inflammation and airway hyperreactivity demonstrated that the deletion of TGF-β receptor 2 decreased the numbers of Th2, Th9, and Th17 cells while increasing Treg cell populations^27^. In the present study, VIT led to an increase in IL-9 as well as TGF-β1 and TGF-β2 levels, although IL-4 levels did not exhibit significant variation over time. This may be attributed to the fact that stimulation of naïve T cells with TGF-β in the absence of IL-4 can prompt the development of inducible Treg cells, which have been shown to alleviate experimental allergic asthma following specific immunotherapy and have also been observed in previous investigations of VIT^7^. No significant changes in the concentration of IL-10 were observed two weeks after VIT, although levels did return to those of the control group at 6 and 24 weeks post-treatment. IL-10 is a potent immunosuppressive cytokine that affects the activity of many different immune cells^28^. The levels of Th-17-related cytokines (IL-1β, IL-6, IL-17A), which are associated with TGF-β signaling, were also unaffected by VIT.

In the present work, changes in the levels of two growth factors, platelet-derived growth factor (PDGF) and granulocyte colony-stimulating factor (G-CSF), were also observed. The role of PDGF in allergies is not well understood, but it has been shown to play a role in the proliferation and migration of airway smooth muscle cells, the enhancement of collagen synthesis by lung fibroblasts, and the polarization and neovascularization of macrophages in asthma^29,30^. PDGF may also be secreted by immune cells (such as mast cells or eosinophils) and epithelial cells, and it has been suggested to affect the complement system. Previous studies have also found a significant increase in the concentration of C3 and C5 in the blood after VIT^7^. The present study is the first to examine the effects of VIT on G-CSF, and it was found that there was a significant decrease in the concentration of the cytokine in the examined group compared to the control group.

In conclusion, the cytokine network has the ability to modulate the immune response patterns following antigen exposure. These alterations are associated with modifications in T helper cells, which play a pivotal role in regulating immune responses. During the long-term response to venom immunotherapy, two main pathways, namely Th1 and Th9, are involved in the modulation of B-cell responses. Among these pathways, the involvement of IL-9 and TGF-β signaling is particularly significant.

## Data Availability

The datasets used and/or analyzed during the current study are available from the corresponding author upon reasonable request.
